# Design and validation of a multiplex PCR method for the simultaneous quantification of *Clostridium acetobutylicum*, *Clostridium carboxidivorans* and *Clostridium cellulovorans*

**DOI:** 10.1038/s41598-023-47007-w

**Published:** 2023-11-16

**Authors:** Laura Feliu-Paradeda, Sebastià Puig, Lluis Bañeras

**Affiliations:** 1 Molecular Microbial Ecology Group, Institute of Aquatic Ecology, University of Girona, Carrer Maria Aurèlia Capmany 40, 17003 Girona, Spain; 2 LEQUiA, Institute of the Environment, University of Girona, Carrer Maria Aurèlia Capmany 69, 17003 Girona, Spain

**Keywords:** Biotechnology, Microbiology, Molecular biology

## Abstract

Co-cultures of clostridia with distinct physiological properties have emerged as an alternative to increase the production of butanol and other added-value compounds from biomass. The optimal performance of mixed tandem cultures may depend on the stability and fitness of each species in the consortium, making the development of specific quantification methods to separate their members crucial. In this study, we developed and tested a multiplex qPCR method targeting the 16S rRNA gene for the simultaneous quantification of *Clostridium acetobutylicum, Clostridium carboxidivorans* and *Clostridium cellulovorans* in co-cultures. Designed primer pairs and probes could specifically quantify the three *Clostridium* species with no cross-reactions thus allowing significant changes in their growth kinetics in the consortia to be detected and correlated with productivity. The method was used to test a suitable medium composition for simultaneous growth of the three species. We show that higher alcohol productions were obtained when combining *C. carboxidivorans* and *C. acetobutylicum* compared to individual cultures, and further improved (> 90%) in the triplet consortium. Altogether, the methodology could be applied to fermentation processes targeting butanol productions from lignocellulosic feedstocks with a higher substrate conversion efficiency.

## Introduction

Anaerobic clostridia are able to convert multiple feedstocks fermentatively into products of economic interest which give them great value for the biotechnological industry^1^. One such product which has gained a lot of attention is biofuels, which can be produced by solventogenic clostridia through the acetone-butanol-ethanol (ABE) fermentation process^2,3^. ABE fermentation has been studied from the early 1920s, when there was an urgent need for acetone, leading to the development of industrial-scale processes such as the Weizmann process^4,5^. However, further progress and development were curtailed due to reported incidents of bacteriophage infections that decreased solvent yields^6^, the easy access to fossil fuels stocks as energy sources, and the improvement of chemical transformations that significantly reduced ABE exploitation^5^. Recently, there has been renewed interest in biological ABE fermentation in the context of preventing climate change effects by reducing net CO_2_ emissions, and to address the energy crisis anticipating lower stocks of fossil fuels^1,2,5^.

One of the latest improvements in fermentation is the replacement of mono-specific bacterial fermentations by artificially designed co-cultures, aimed at increasing substrate spectrum and improve the product yield^5,7–9^. Co-cultures of *Clostridium* spp., particularly combining cellulolytic (e.g., *C. thermocellum*) and solventogenic strains (e.g., *C. thermosaccharolyticum, C. beijerinckii, C. acetobutylicum*) have been used previously^5,7,8,10^. Similarly, co-cultures of *C. cellulovorans* and *C. acetobutylicum* have been tested by Valdez-Vazquez et al.^11^ as the desired combination to target increased hydrogen production from lignocellulosic (wheat straw) sources. The consortium offers dual benefits and reveals tight metabolic dependency for increased productivities, as *C. acetobutylicum* grows rapidly using different sugars to high optical densities, while *C. cellulovorans* produces large quantities of acids (i.e., acetic and butyric acid) and CO_2_ and H_2_ as fermentation end-products^12,13^. A recent survey on butanol production with *C. acetobutylicum* revealed the addition of exogenous butyric acid as a determinant factor^14^. Therefore, co-cultures of *C. cellulovorans* and *C. acetobutylicum*, may lead to improved butanol production after intense butyric acid production at early stages of fermentation. To further improve the process, an acetogenic strain able to grow and produce acids and alcohols from CO_2_ and H_2_ could be used in a mixed culture. Among acetogenic *Clostridium* species, *C. carboxidivorans* is of particular interest as it can convert CO_2_ and CO into acetic acid and ethanol as well as higher carbon compounds such as butanol or hexanol^15–17^.

In spite of significant progress compared to mono-cultures, artificial co-cultures usually resolve in one species out-competing the other, leading to unpredictable consortium dynamics^18^, which negatively affect production processes in long fermentation runs. To address this issue, specific monitoring of consortium members is necessary and should be carefully considered to gain knowledge of the individual growth kinetics if the optimization of the fermentation process is targeted^18,19^. Traditional methods to monitor the growth include optical density, cell dry weight, cell count and protein content measurements. However, most of them cannot discriminate between species when used in co-cultures. In this sense, molecular-based techniques such as real-time quantitative PCR (qPCR) or Fluorescence in situ hydration (FISH) have been used as alternatives due to their discrimination capacity, high sensitivity and robustness, despite requiring longer protocols. While FISH allows the observation of cell morphology changes and possible associations between cells^20^, it is time-consuming when used as a quantitative method. In contrast, qPCR can provide reliable quantitative measurements of distinct cell species at high speed, and can be easily automated. Nevertheless, qPCR methods cannot discriminate between life and dead cells^21,22^.

Several qPCR assays have been used to target *Clostridium* species^22–26^. Among them, qPCR coupled with double-labelled TaqMan probes designed to be species-specific allows accurate detection of consortium members in a single reaction^27^. Multiplex qPCR using TaqMan probes has been implemented for rapid and specific detection of different *Clostridium* species, mainly in the food industry^22,23,28^. However, few examples exist of species-specific probes designed to monitor fermentation processes involving solventogenic clostridia. For instance, Lee et al.^25^ designed primer and probe sets targeting the pSOL1 plasmid and the 16S rRNA gene of *C. acetobutylicum* to control the loss of butanol production capacity during ABE fermentation. Jiang et al.^29^ implemented a co-culture of *C. thermocellum* and *T. pseudoethanolicus* to continuously ferment cellulose into bioethanol, and species-specific probes targeting the 16S rRNA gene were designed to monitor the growth dynamics of both species. Therefore, it is crucial to develop and implement species-specific probes for monitoring artificial co-cultures of *Clostridium* spp. to achieve stable and optimized fermentation processes.

The purpose of this study was to develop a triplex quantitative PCR methodology using a single primer pair and three species-specific TaqMan probes targeting the 16S rRNA gene of three *Clostridium* species that are known to provide significant advantages when used in co-culture. The probes were tested in different consortium simulations both as extracted DNA or cell densities. Additionally, product yields from cellobiose and glucose were evaluated. A cellulolytic, *C. cellulovorans* 743B*,* a solventogenic, *C. acetobutylicum* ATCC824, and an acetogenic, *C. carboxidivorans* P7, strains were used as model organisms for fermentation purposes. To the best of our knowledge, no previous studies on ABE fermentation combining the three *Clostridium* species used in this work have been done, nor have any species-specific TaqMan probes been developed for the detection of *C. cellulovorans* nor *C. carboxidivorans*. In this sense, this research not only involved the development of a monitoring methodology for the simultaneous quantification of three *Clostridium* species but also an assessment of the alcohol production obtained using artificially mixed cultures in comparison to respective monocultures.

## Results

### Efficiency, quantification range and detection limits of qPCR assays

**Figure 1:**
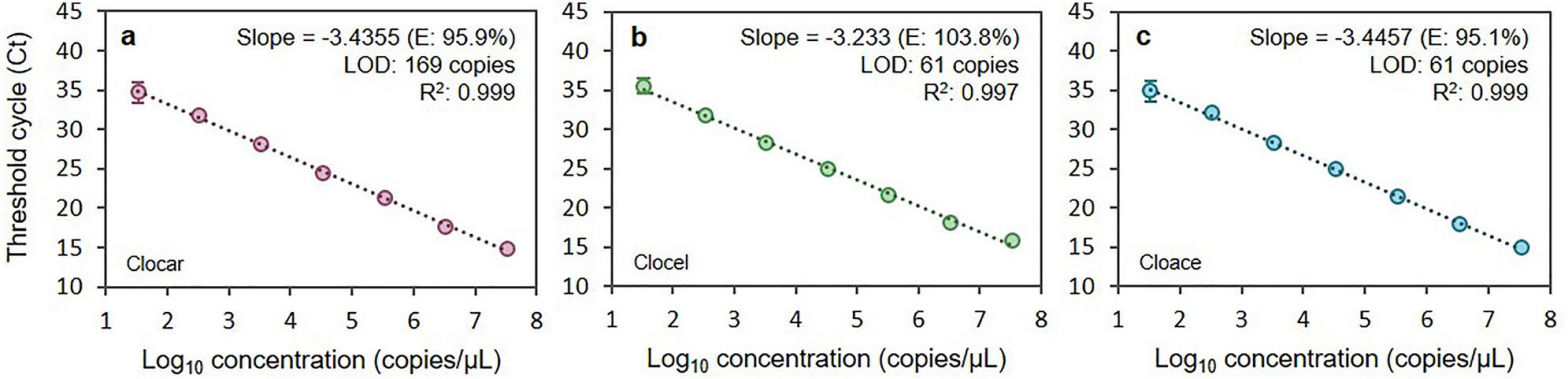
Standard curves obtained for (**a**) Clocar, (**b**) Clocel and (**c**) Cloace probes from C_t_ and log_10_ value of copy number in tenfold serial dilutions. N = 10, error bars ± SD.

To determine the parameters of the multiplex quantitative PCR methodology, three standard curves were tested in a range from 3.33 × 10^1^ to 3.33 × 10^7^ gene copies/µL (Fig. 1). The quantification range for the three probes was linear, with no deviations between replicates, spanning 6 log units from 3.33 × 10^2^ to 3.33 × 10^7^ gene copies. Standard curves within this range exhibited excellent reaction efficiencies: 95.1% for *C. acetobutylicum* probe (Cloace, b = − 3.4457); 95.9% for *C. carboxidivorans* probe (Clocar, b = − 3.4355); and 103.8% for *C. cellulovorans* probe (Clocel, b = − 3.233). The detection limit (LOD) at a confidence level of 95% was determined to be 61.24 copies for both Cloace and Clocel probes and 168.67 copies for Clocar, while the quantification limit (LOQ) within the linearity range was of 333 copies for all probes.

A verification of the multiplex qPCR assay with OD readings was performed (Supplementary Fig. S1). A good linear correlation could be found between the two methodologies, indicating the good performance of the multiplex qPCR for determining the growth of these *Clostridium* species.

### Assessing the specificity of TaqMan probes for the detection of *Clostridium* species

To check for probe specificity, DNA extracts from ten different *Clostridium* strains and four other *Firmicutes* that were available in our laboratory were tested for amplification (Table 1). Clocel and Cloace probes were highly specific and showed no amplification signal in the absence of the target DNA. However, the Clocar probe showed an intense positive amplification signal for *Clostridium drakei* and a faint amplification signal for *C. ljungdahlii* and *C. autoethanogenum*. The 16S rRNA gene sequences of the four species are high similar in the region PCR primers and TaqMan probes were directed. Forward and reverse PCR primers contained one and three mismatches to *C. ljungdahlii/ C. autoethanogenum* sequence, respectively, and Clocar probe contained a single mismatch to the *C. ljungdahlii/ C. autoethanogenum* sequence (Supplementary Fig. S2).

**Table 1:** PCR specificity tests of each TaqMan probe using a total of 14 different bacterial DNA extracts.

Organism	PCR amplification		
	Clocar	Clocel	Cloace
*C. acetobutylicum* DSM 792^T^	−	−	+
*C. carboxidivorans* DSM 15243^T^	+	−	−
*C. cellulovorans* DSM 3052^T^	−	+	−
*C. kluyveri* DSM 555^T^	−	−	−
*C. drakei* DSM 12750^T^	+	−	−
*C. leptum* DSM 753^T^	−	−	−
*C. autoethanogenum* DSM 10061^T^	±	−	−
*C. ljungdahlii* DSM 13528^T^	±	−	−
*C. viride* DSM 6836^T^	−	−	−
*C. coccoides* ATCC 29236^T^	−	−	−
*Eubacterium limosum* DSM 20543^T^	−	−	−
*Lactobacillus plantarum* ATCC 14917^T^	−	−	−
*Leuconostoc mesenteroides* ATCC 8293^T^	−	−	−
*Staphylococcus aureus* DSM 20231^T^	−	−	−

+ clear amplification detected; ± weak amplification detected; − no detected amplification.

The designed quantitative PCR aimed to investigate the growth of the three strains individually in the course of fermentation experiments with combinations of synthetic consortia. The number of cells per mL was estimated from the number of gene copies per mL taking into account the number of 16S rRNA gene copies present in *Clostridium* genomes (9 copies for *C. cellulovorans*, and 11 for *C. acetobutylicum* and *C. carboxidivorans*) according to the information available in the whole genome sequence (GenBank accession numbers CP002160, AE001437 and CP011803, respectively). Since the three strains may exhibit different growth rates, their proportions can vary at different time point during fermentation. Consequently, the specificity of the qPCR was tested in synthetic mixtures of *C. cellulovorans, C. acetobutylicum* and *C. carboxidivorans,* at concentrations of 0.1, 1.0 and 10 ng of genomic DNA/µL. By using the triplex qPCR assay, we successfully detected the three different *Clostridium* species when mixed at different proportions, without signals of inhibition in the range assayed from 1.36 × 10^8^ ± 1.09 × 10^8^ cells/mL (10 ng/µL extracted DNA) to 1.27 × 10^6^ ± 7.91 × 10^5^ cells/mL (0.1 ng/µL).

### Influence of *C. ljungdahlii* and *C. autoethanogenum* in Clocar probe targeting *C. carboxidivorans*

**Figure 2:**
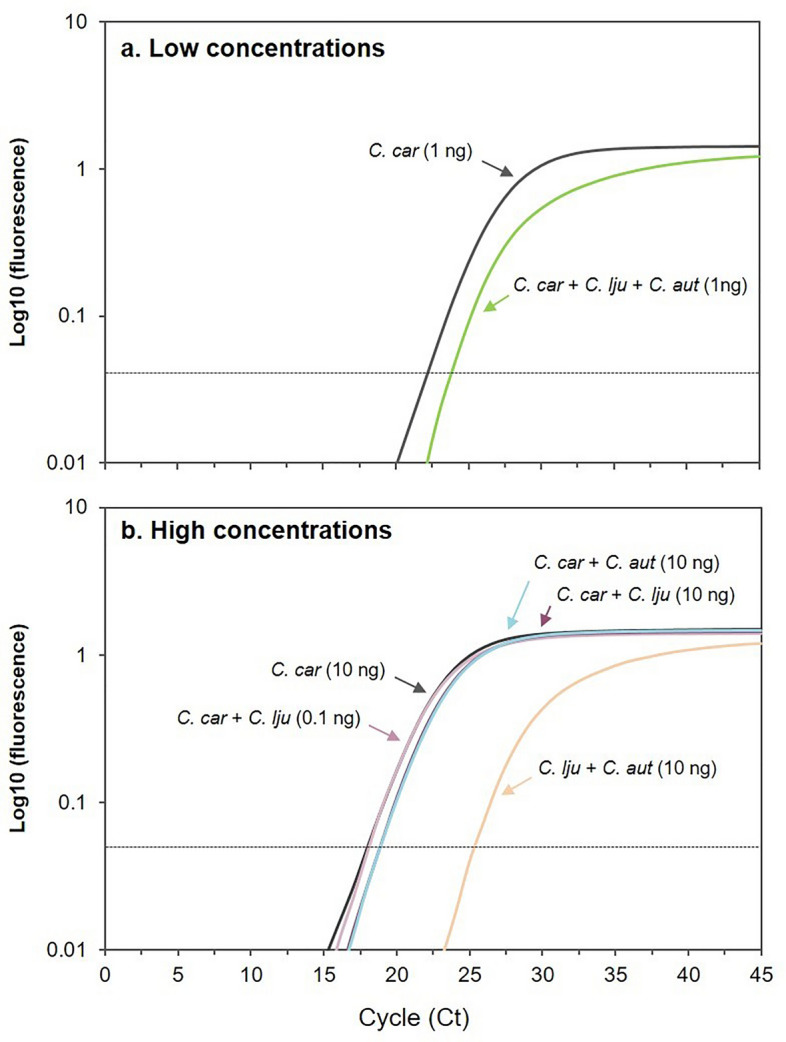
Amplification curves of the artificial DNA mixtures both at low (**a**) and high (**b**) concentrations. (**a**) Curves shown were obtained when using 1 ng of pure *C. carboxidivorans* (in black, used as a reference) alone and when mixed with 1 ng of *C. ljungdahlii* and 1 ng of *C. autoethanogenum* (green). (**b**) At high concentrations, curves showed were obtained when using 10 ng of pure *C. carboxidivorans* (in black, used as reference), when mixed with different amounts of *C. ljungdahlii* (pink) or *C. autoethanogenum* (blue), or without *C. carboxidivorans* only containing 10 ng of both *C. ljungdahlii* and *C. autoethanogenum* (orange). The dotted line indicates the threshold cycle number.

To further investigate the influence of *C. autoethanogenum* and *C. ljungdahlii* on the performance of the Clocar probe, synthetic DNA mixtures were prepared at two concentration ranges: 1 ng (low) or 10 ng (high) of genomic DNA/µL of *C. carboxidivorans,* along with *C. ljungdahlii* and *C. autoethanogenum* (Fig. 2). The mixing of 1 ng of DNA of *C. ljungdahlii* and *C. autoethanogenum* together with 1 ng of DNA of *C. carboxidivorans* resulted in a slight underestimation the later (107.4%) (Fig. 2a). At higher concentrations of *C. carboxidivorans* (10 ng of genomic DNA/µL), the presence of 10 ng of DNA of *C. ljungdahlii* or *C. autoethanogenum* had a relatively smaller effect (104.5 to 105.1% change, respectively) (Fig. 2b). In the absence of *C. carboxidivorans*, Clocar probe still exhibited amplification when 10 ng of both *C. autoethanogenum* and *C. ljungdahlii* were present, quantifying an abundance of near 0.3 ng/µL of *C. carboxidivorans* even though this species was absent in the mixture (Fig. 2b), further reaffirming that Clocar probe cannot clearly discriminate between the three species likely due to the high similarity of the 16S rRNA gene between *C. carboxidivorans* and *C. ljungdahlii / C. autoethanogenum.* Although the proposed tool is reliable for its use for the three selected species, it may cause some deviations if microorganisms such as *C. ljungdahlii / C. autoethanogenum* are used in combination with *C. carboxidivorans.* In this situation, methods targeting functional genes as those proposed earlier should be used^26^.

### Monitoring of growth dynamics in synthetic consortia

**Figure 3:**
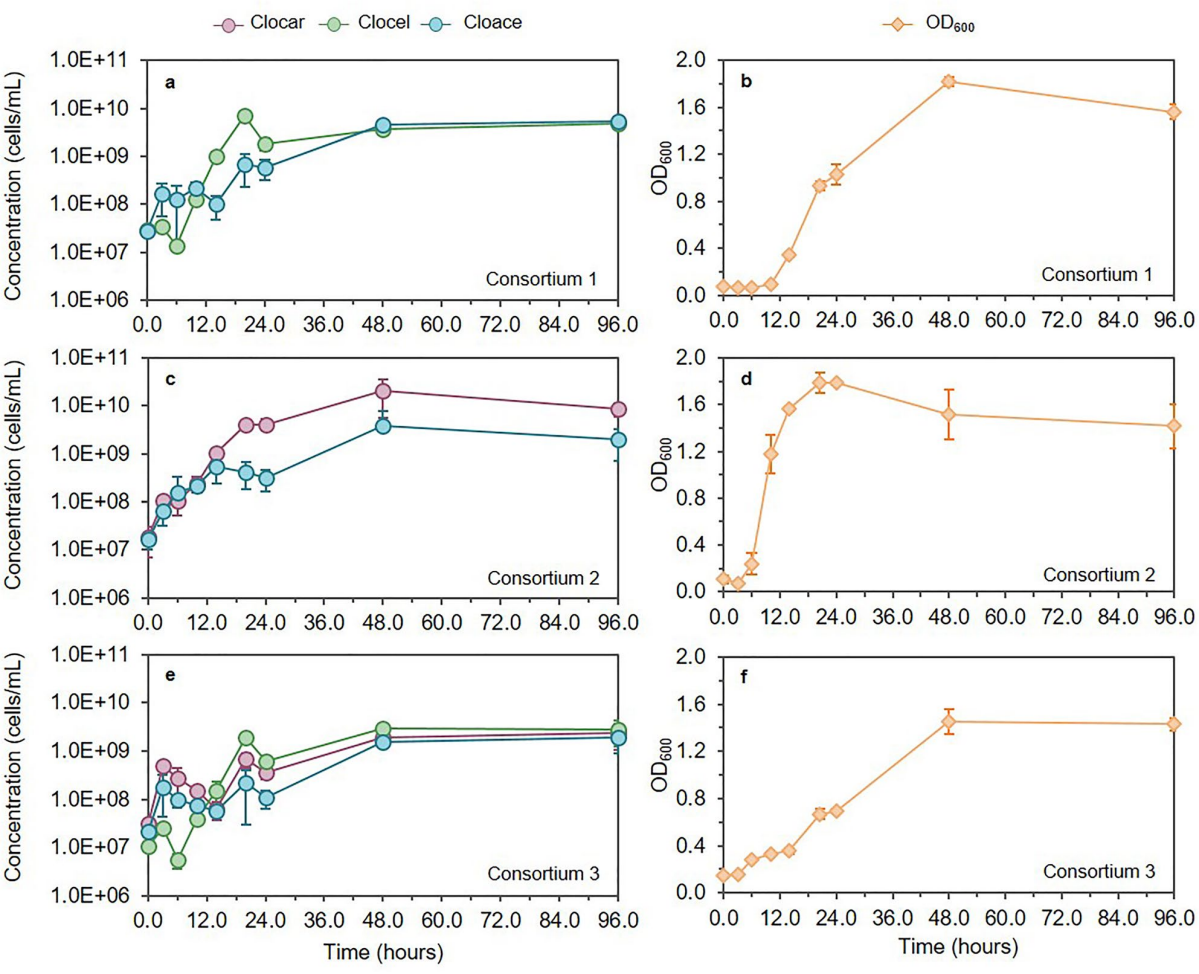
*C. acetobutylicum* (Cloace), *C. carboxidivorans* (Clocar) and *C. cellulovorans* (Clocel) growth curves within three different consortia (1, 2 and 3) in a 4-day batch experiment in batch reactors. Note the logarithmic scale on the y axes. N = 3, error bars ± SD. Carbon source: 5 g/L of cellobiose in consortia 1 and 3; 5 g/L of glucose in consortia 2.

In order to evaluate the effectiveness of the methodology using broth from real fermentation experiments, the triplex qPCR assay was applied on three different consortia implemented by combining the three *Clostridium* species. These consortia were operated in anaerobic flasks at 37 °C for a 4-day batch fermentation period, with triplicates for each set. A new medium composition useful for the three strains was initially designed. Mineral components were defined as a combination of the three DSMZ recommended media, and 5 g/L of cellobiose (consortia 1 and 3) or glucose (consortium 3) were chosen as the carbon source (see “Materials and Methods” section). This newly defined medium was tested for growth of the three strains individually resulting in similar results compared to the DSMZ recommended medium. Thorough the entire batch fermentation process, all consortia could be consistently detected and quantified, and TaqMan probes only showed amplification in the presence of the target DNA (Fig. 3). In consortia 1, *C. cellulovorans* exhibited immediate growth after inoculation reaching the stationary phase after 20 h, whereas *C. acetobutylicum* showed a longer lag phase and started growing progressively after 14 h of inoculation. A similar dynamic was found in consortium 3, in the presence of *C. carboxidivorans*. On the other hand, both *C. acetobutylicum* and *C. carboxidivorans* in consortium 2 exhibited simultaneous growth without any observable lag phase. Growth lasted for 48 h before reaching the stationary phase. Notably, *C. acetobutylicum* concentration in this consortium was 1-log lower in comparison to *C. carboxidivorans*. Although the growth patterns among the strains were different, it was apparent they were able to coexist throughout the entire experimental period. Nevertheless, in almost all cases, a decrease in the maximum growth rate of the three microorganisms was observed when compared to their growth as pure cultures in the same conditions (Table 2). Among the three species, *C. cellulovorans* appeared to be the less affected by the presence of the other species*.*

**Table 2:** Growth rate (h^−1^), biomass yield (in cells per gram of carbon source), and product yields (grams of product per gram of carbon source) of the three Clostridium species grown as mono-cultures or in different combinations.

Culture type	*Clostridium* species	Growth parameters		Product yield (g/g of carbon source)					
		µ (h^−1^)	Y_X/S_ (cells/g)	Y_EtOH_	Y_AH_	Y_BuOH_	Y_BH_	Y_Acetone_	Y_Others_
Mono-cultures	*C. carboxidivorans*_(glu)_	0.78 ± 0.25	3.3 × 10^9^ ± 4.7 × 10^8^	0.090 ± 0.024	0.100 ± 0.010	0.003 ± 0.001	0.012 ± 0.001	–	0.003 ± 0.001
	*C. carboxidivorans*_(ce)_	0.20 ± 0.02	Not determined						
	*C. acetobutylicum*_(glu)_	0.66 ± 0.01	4.3 × 10^8^ ± 1.5 × 10^8^	0.009 ± 0.003	0.117 ± 0.025	0.011 ± 0.004	0.279 ± 0.055	0.005 ± 0.001	–
	*C. acetobutylicum*_(ce)_	0.43 ± 0.09	Not determined						
	*C. cellulovorans*_(ce)_	0.66 ± 0.03	2.6 × 10^9^ ± 4.6 × 10^8^	–	0.049 ± 0.006	–	0.162 ± 0.031	–	–
Consortia 1_(ce)_	*C. acetobutylicum*	0.18 ± 0.03	1.8 × 10^9^ ± 3.6 × 10^8^	0.008 ± 0.002	0.114 ± 0.011	0.009 ± 0.003	0.303 ± 0.041	0.002 ± 0.000	–
	*C. cellulovorans*	0.54 ± 0.02							
Consortia 2_(glu)_	*C. acetobutylicum*	0.28 ± 0.19	2.3 × 10^9^ ± 8.9 × 10^8^	0.106 ± 0.014	0.149 ± 0.018	0.033 ± 0.011	0.111 ± 0.005	0.002 ± 0.001	0.023 ± 0.006
	*C. carboxidivorans*	0.29 ± 0.02							
Consortia 3_(ce)_	*C. acetobutylicum*	0.09 ± 0.08	1.2 × 10^9^ ± 6.2 × 10^8^	0.035 ± 0.004	0.079 ± 0.003	0.095 ± 0.003	0.068 ± 0.014	0.001 ± 0.000	0.026 ± 0.003
	*C. cellulovorans*	0.41 ± 0.07							
	*C. carboxidivorans*	0.20 ± 0.08							

The carbon source used in the medium is marked as glu- (glucose), or ce- (cellobiose) in brackets next to the calculated value. Caproic acid and hexanol are grouped and represented as Others. N = 3, ± SD.

*EtOH* ethanol, *AH* acetic acid, *BuOH* butanol, *BH* butyric acid.

### Alcohols and organic acids production by mono-cultures and consortia

**Figure 4:**
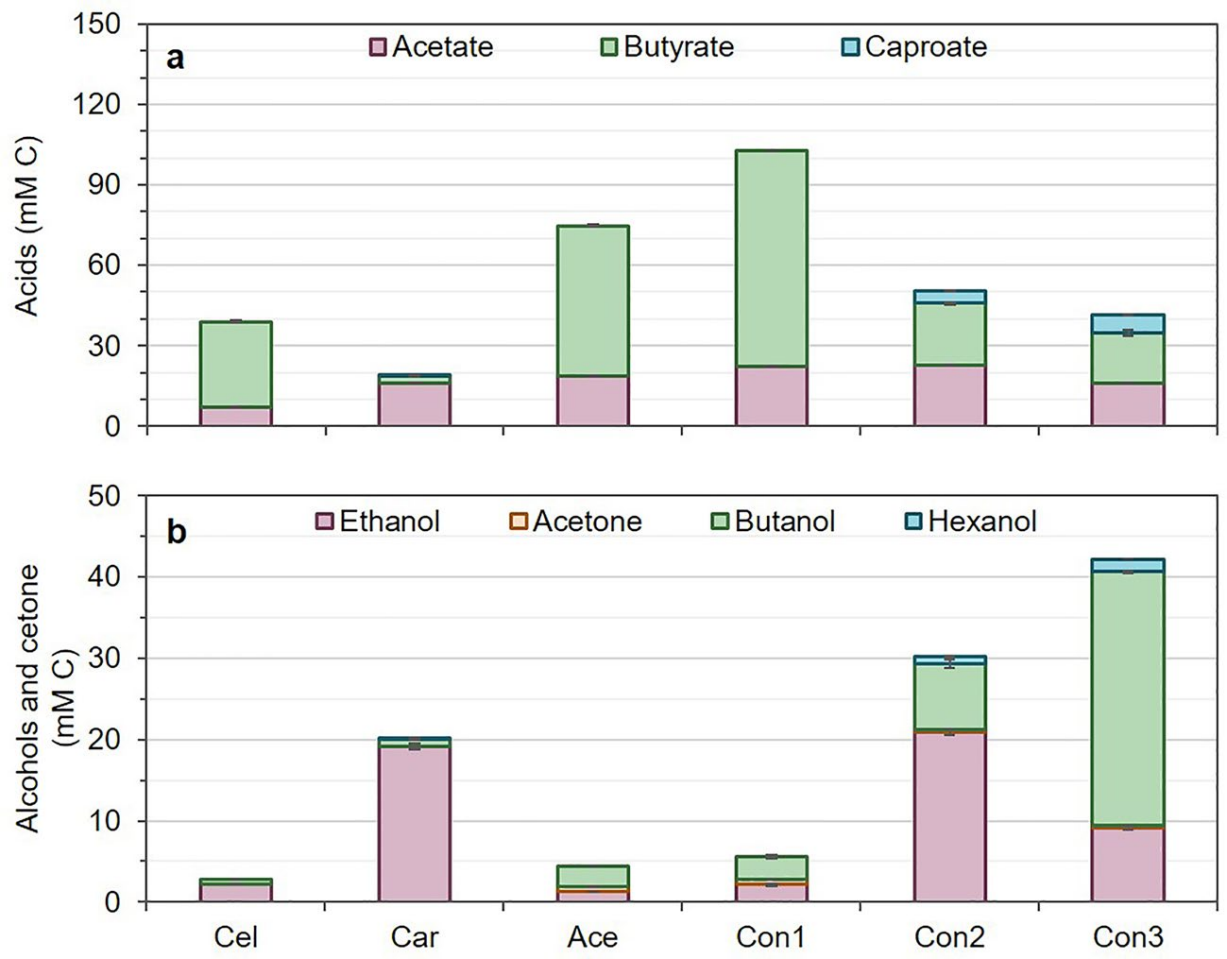
Acids (**a**) and alcohols and acetone (**b**) concentrations in mmols per L of carbon (mM C) produced by the three mono-culture and consortia 4-day fermentation experiment. Note Y-axis differ in alcohols and acids. Cel: *C. cellulovorans*; Car*: C. carboxidivorans*; Ace: *C. acetobutylicum*; Con1: consortium 1; Con2: consortium 2; Con3: consortium 3. Carbon source used in consortium 2 and *C. carboxidivorans* and *C. acetobutylicum* mono-cultures: 5 g/L glucose; carbon source used in consortia 1 and 3 and *C. cellulovorans* mono-culture: 5 g/L cellobiose. In all cases, the initial gas phase was a mixture of 20% CO_2_ and 80% H_2_. Concentration values for each compound can be found in Supplementary Table S1.

The production of organic acids and alcohols (Fig. 4, Supplementary Figs. S3 and S4) and growth rates (Table 2) significantly differed between cultures and carbon source. As anticipated, when mono-cultures were used, *C. carboxidivorans* predominantly produced acetic acid (AH, 16.25 ± 1.82 mM C, 14.21% of transformed C) at a yield of 0.10 ± 0.01 g AH g S^−1^*.* Conversely*, C. acetobutylicum* and *C. cellulovorans* exhibit butyric acid (BH) as the most abundant carboxylic acid (55.94 ± 5.12 and 31.78 ± 6.06 mM C, production yields of 0.28 ± 0.06 and 0.16 ± 0.03 g BH g S^−1^, respectively). Caproic acid was only found as residual concentrations in *C. carboxidivorans.* Ethanol (EtOH) was the main alcohol produced by *C. carboxidivorans* and *C. acetobutylicum.* Ethanol to acetic acid reached an almost 1:1 ratio in *C. carboxidivorans* when grown on glucose. Butanol was detected as a minor alcohol in *C. carboxidivorans* and *C. cellulovorans,* but notably increased, along with acetone, in *C. acetobutylicum* (2.59 ± 0.65 and 0.62 ± 0.22 mM C, respectively). *C. acetobutylicum* predominantly produced both butyric and acetic acids (55.94 ± 2.59 and 18.75 ± 1.70 mM C, respectively) following a ratio similar to that observed in typical ABE fermentation.

Analyzing the different consortia, the production of alcohols and organic acids exhibited noteworthy changes compared to pure strains. Consortium 1 (*C. cellulovorans* and *C. acetobutylicum*) yielded the highest production of acids (80.5 ± 1.36 and 22.36 ± 1.24 mM C of butyric and acetic acids, respectively), and the highest butyric acid production yield (0.30 ± 0.04 g BH g S^−1^). However, the overall production of alcohols remained low in this consortium (< 5 mM C). This was probably due to the higher estimated growth rate of *C. cellulovorans* (0.54 ± 0.02 h^−1^) compared to *C. acetobutylicum* (0.18 ± 0.03 h^−1^) at the start of the experiment. *C. cellulovorans* produces only acids from cellobiose. On the other hand, consortium 2 (*C. carboxidivorans* and *C. acetobutylicum*) and 3 (all three species) showed a higher production of solvents whereas production of carboxylic acids was reduced if compared to consortium 1. Caproic acid and hexanol were specifically detected in consortia 2 and 3 (~ 5 mM C and 1 mM C, respectively). Acetone remained at low concentrations, being 0.60 ± 0.10 mM C (< 1% of transformed C). The highest butanol production (31.27 ± 0.57 mM C, 0.10 ± 0.00 g BuOH g S^−1^) was obtained in consortium 3.

### Reliability of TaqMan probes quantification versus general established methods

**Figure 5:**
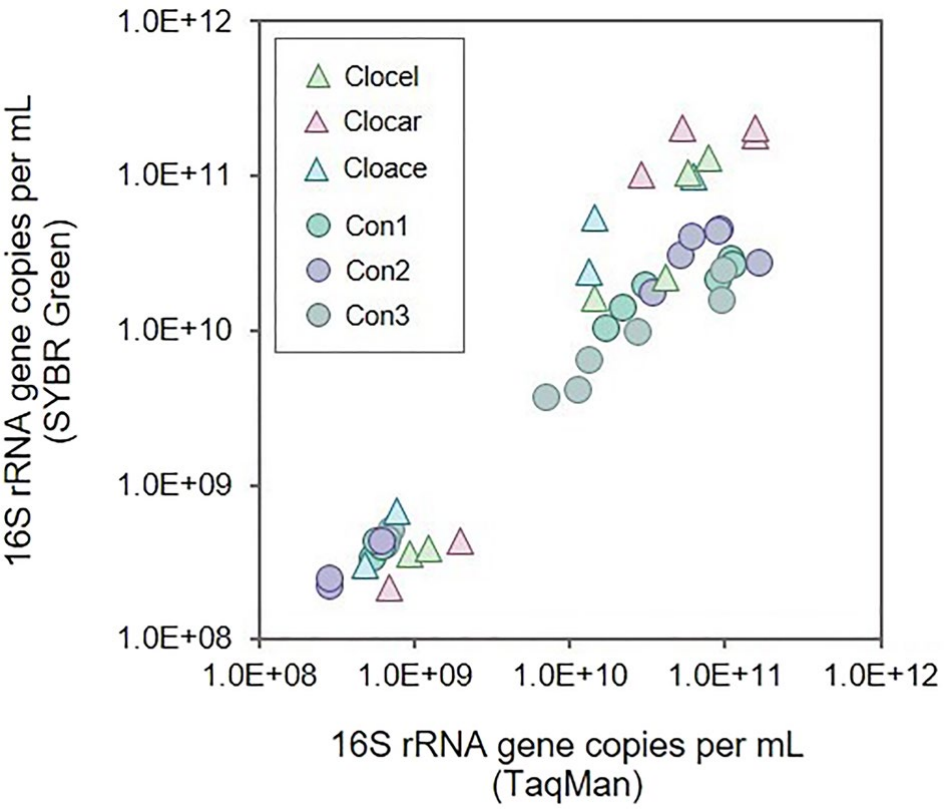
16S rRNA gene concentration (copies per mL) obtained using TaqMan probes (X-axis) plotted against the concentration obtained using SYBR Green with the universal bacterial primer pair 341F–534R (Y-axis).

Quantitative PCR using SYBR Green and the primer pair 341F–534R specific for *Eubacteria* were also tested using samples obtained from consortia and pure cultures. We used this procedure to validate the accuracy of the TaqMan probes and to check for the presence of an eventual contamination during fermentation. The number of 16S rRNA gene copies per mL determined using this qPCR assay, was plotted against the number of 16S rRNA gene copies per mL detected through the triplex qPCR assay (Fig. 5). The results revealed that gene concentrations in the samples were similar when analysed with both qPCR assays, even though consortia showed slightly higher concentrations when TaqMan probes were employed.

To discard whether the discrepancy between the two methods was attributable to contamination, barcoded amplicon sequencing of the 16S rRNA gene was used. Although the sequencing method may have some drawbacks due to a reduced detection capacity of very low-represented phylotypes, it could provide valuable insights of an important contamination event. In the different consortia samples, all retrieved sequence reads matched (> 99.5%, ~ 250 bp fragment) to the 16S rRNA gene sequence of the three species used (Supplementary Fig. S5), suggesting that no contamination existed. Therefore, the slight differences observed between the two quantification methods are likely a result of undesired effects in the PCR reactions.

## Discussion

In this study, we developed a triplex qPCR method to quantify three different model *Clostridium* species (*C. cellulovorans* 743B*, C. acetobutylicum* ATCC 824 and *C. carboxidivorans* P7) commonly used in fermentation of sugars. The methodology effectively differentiated between the three Clostridia in both synthetic DNA mock communities (implemented by mixing DNA extracts mimicking different concentration proportions), and in real consortia under laboratory conditions.

Our approach involved targeting a specific region of the 16S rRNA gene using TaqMan probes allowing for a simplified and unified PCR reaction for the three tests. PCR primers were designed targeting two consensus regions flanking a short variable region where TaqMan probes were directed. The quantification of the 16S rRNA gene served as a proxy for cell quantification which holds relevant for biotechnological applications^24,30,31^. Notably, the estimation of cell numbers is only feasible if the exact number of 16S rRNA gene copies is known for a given organism^32^. The designed Clocel and Cloace probes exhibited high specificity for their respective target organism (*C. cellulovorans* and *C. acetobutylicum*, respectively) with no interferences observed from other DNAs. In addition, the quantification method was highly efficient (103.8% and 95.1%, respectively). In contrast, Clocar probe did not show unspecific amplifications of *C. cellulovorans* and *C. acetobutylicum*, but was not suitable to completely discriminate other closely related species, such as *C. drakei* (99.6% similarity of the 16S rRNA gene with *C. carboxidivorans*)*,* and other similar strains such as *C. autoethanogenum* and *C. ljungdahlii* (94% similarity of the 16S rRNA gene with *C. carboxidivorans*)^33,34^. For instance, these acetogenic species do not only share similar 16S rRNA gene similarities, but have significant similarities along their genome sequences, despite differing in key aspects of their physiology which are used to distinguish between these species (such as the ethanol production capacity in the case of *C. autoethanogenum* and *C. ljungdahlii*^26,35^, or the capability to produce butanol that *C. carboxidivorans* has since it encodes butanol dehydrogenase which the other acetogenic strains do not possess^33,36^).

When testing the methodology in laboratory cultures, we detected and quantified all three *Clostridium* species during batch fermentations. Moreover, total gene concentrations (addition of the three values) from our triplex qPCR assay matched those values obtained with a regular qPCR detection method using 16S rRNA gene targeting pair 341F–534R^37^, thus reinforcing the reliability of the proposed quantification method. We confirmed the co-existence of the three *Clostridium* species (either as tandem cultures or a triplet consortium) in batch fermentation thus allowing to determine their individual growth rate (Table 2). Altogether, in silico tests as well as quantitative PCR performed using both synthetic DNA mixtures and real implemented consortia revealed that Clocar and specially Clocel and Cloace probes could clearly quantify with high specificity *C. carboxidivorans, C. cellulovorans* and *C. acetobutylicum,* pointing out their potential application for the identification of these Clostridia in bioreactor samples.

Although our primary objective was not to optimize alcohol production in mixed cultures, we analyzed how the combination of different species could affect the acid-to-alcohol ratio in batch fermentations. The analysis revealed that consortia, compared to mono-cultures, exhibited increased production profiles of both acids and alcohols. In this sense, the combination of *C. acetobutylicum* with *C. cellulovorans*, which cannot produce alcohols from the fermentation of sugars (i.e., cellobiose)^12,13^, did not show any improvement in alcohol production compared to mono-cultures. Contrarily, when *C. acetobutylicum* was co-cultured with *C. carboxidivorans* (consortium 2), or in a triplet consortium with also *C. cellulovorans* (consortium 3), alcohol production was highly boosted compared to mono-cultures. Both *C. acetobutylicum* and *C. carboxidivorans* have the ability to produce ethanol and butanol from simple sugars^2,5,38^. In consortium 2, both species exhibited exponential growth during the first hours (Fig. 3) converting glucose into acetic and butyric acids and causing a drop in the pH which shifted both *C. carboxidivorans* and *C. acetobutylicum* metabolisms from acidogenesis to solventogenesis^38,40^, since ethanol and butanol were promptly found (Supplementary Fig. S4). Furthermore, the implementation of a triplet consortium with the three *Clostridium* species significantly increased solventogenesis since alcohol production equaled that of acids (alcohol to acid ratio of 1.01). Moreover, consortium 3 produced the highest butanol concentration of all tested conditions, with a four- and ten-fold increase in comparison to consortium 2 and consortium 1 or mono-cultures, respectively. In this consortium, growth right after inoculation of *C. cellulovorans* produced high butyric acid concentrations and decreased pH, which shifted *C. acetobutylicum* and *C. carboxidivorans* metabolism to solventogenesis. This switch into solventogenesis led to a higher re-assimilation of butyric acid, and therefore its conversion into butanol^38,40^ or its elongation into C6 compounds by *C. carboxidivorans* that, as already reported in other studies^15,16,39,41^, has the ability to produce caproic acid and hexanol through hexanol-butanol-ethanol (HBE) fermentation from simple sugars or gaseous substrates. Since CO_2_ and H_2_ were used in the gas phase to maintain anaerobic conditions at the start-up of fermentation runs, an effective use of these substrates by *C. carboxidivorans* cannot be ruled out. Nevertheless, productions increased significantly compared to monocultures thus confirming produced CO_2_ and H_2_ could be further used in the mixed consortia.

Butanol production using syntrophic or mixed cultures can be largely competitive towards mono-culture fermentation^10^. As reviewed in Pinto et al.^10^, since they mainly run in batch or fed-batch to avoid contamination, long lag phases and the uncoupling of growth as a result to a poor growth adaptation of the syntrophic partner constitute the limitations of syntrophic co-cultures. Our findings shed light on the impact of mixed cultures on butanol production and the acid-to-alcohols ratio. The use of a triplex qPCR assay was implemented for a rapid and simultaneous quantification of three model *Clostridium* species involved in butanol production, cellulose degradation and syngas fermentation (*C. acetobutylicum, C. cellulovorans,* and *C. carboxidivorans*, respectively). This methodology was proven effective in synthetic co-cultures, enhancing our understanding on the acid to alcohol profiles obtained since it specifically determined each species growth dynamics within the different consortia. Consequently, it holds promise for targeted analysis and optimization of these species in mesophilic bioreactors targeting butanol production from a cellulosic feedstock.

## Materials and methods

### Bacterial strains and maintenance conditions

*Clostridium cellulovorans* 743B (DSM 3052), *Clostridium acetobutylicum* ATCC 824 (DSM 792) and *Clostridium carboxidivorans* P7 (DSM 15243) were obtained from Leibniz Institute DSMZ—German Collection of Microorganisms and Cell Cultures (www.dsmz.de). The three strains were cultured in the recommended media according to the DSMZ instructions (*C. cellulovorans* in DSM520 medium, *C. acetobutylicum* in DSM104b medium and *C. carboxidivorans* in DSM104c medium) and incubated in sealed serum tubes (Ochs Gläsgeratebau, Germany) at 37 °C and 130 rpm under anaerobic conditions until cryo-stocks were prepared and frozen at − 80 °C. Cultures were activated from cryo-stocks and maintained active by successive 1/10 transfers into anaerobic tubes containing 10 mL of freshly prepared medium for a maximum of two transfers. Overnight cultures (< 14 h old) of individual species were used as inocula for consortia experiments. Headspace to liquid volume ratio was 1.5:1 v:v, with an initial gas pressure of 0.2 bar (CO_2_:H_2_ or N_2_, 20:80% and 100% respectively).

The consortia mineral medium was designed by mixing the different mineral components from the recommended DSM medium, yeast extract and the corresponding carbon source, and contained in 1L: KH_2_PO_4_, 1 g; K_2_HPO_4_, 1 g; (NH_4_)_2_SO_4_, 1 g; FeSO_4_ × 7H_2_O, 0.001 g; MgCl_2_ × 6H_2_O, 0.2 g; CaCl_2_ × 2H_2_O, 0.075 g; trace element solution SL-10, 1 mL; Na_2_CO_3_, 1.5 g; Na-resazurin, 0.5 mg; l-cysteine × HCl, 0.45 g; yeast extract, 5 g; and 5 g/L of cellobiose or glucose. Cellobiose was used as the substrate for the growth of the cellulose-degrading *C. cellulovorans,* either in pure or mixed cultures. Glucose was used elsewhere. The trace metal solution SL-10 contained, in 1L: HCl 25%, 10 mL; FeCl_2_ × 4 H_2_O, 1.,5 g; ZnCl_2_, 70 mg; MnCl_2_ × 4 H_2_O, 0.1 g; H_3_BO_3_, 6 mg; CoCl_2_ × 6 H_2_O, 0.19 g; CuCl_2_ × 2 H_2_O, 2 mg; NiCl_2_ × 6 H_2_O, 24 mg; Na_2_MoO_4_ × 2 H_2_O, 36 mg. *C. cellulovorans* DSM 3052, *C. acetobutylicum* DSM 792 and *C. carboxidivorans* DSM 15243 were first grown individually in the consortia mineral medium to determine the growth parameters of the three species in mono-cultures.

### Design of primer pair and TaqMan probes

In order to quantify the three different *Clostridium* species simultaneously, a multiplex qPCR assay was designed. The method consisted of a unique universal primer pair targeting the bacterial 16S rRNA gene and 3 specific hydrolysis TaqMan probes targeting the three *Clostridium* species individually. Both, 16S rRNA primers and TaqMan probes were specifically designed. Complete 16S rRNA gene sequences of *C. cellulovorans, C. acetobutylicum* and *C. carboxidivorans* were downloaded from the NCBI (www.ncbi.nlm.nih.gov) genomic database, aligned using Clustal Omega, and conserved regions flanking a more dissimilar region in the 16S rRNA gene sequences were identified as preferential for primer and probes targets.

The primer pair was designed with Primer-BLAST from NCBI, with the following specifications: expected amplicon size was limited to 150 bp; the melting temperature of each primer was set to 58 ± 1 °C and a GC content of 30–80%. Species-specific TaqMan probes were designed following the guidelines provided by Applied Biosystems (hybridization temperature 8–10 °C higher than the primer pair, and a GC content near 40%), and the specificity and self-complementarity of the primer pair and probe sets were further checked using the software Primer Express® v3.0 (Applied Biosystems, CA) while checking for unspecific primer-dimers or primer–probe complementarity. Probe specificity at the species level was verified with BLAST searches optimized for small sequences. Primers were synthesised by Invitrogen (Thermo Fisher Scientific, Spain) and probes were acquired from Biomers (Ulm, Germany).

The resulting primer pair and the three TaqMan probes are listed in Table 3. Clocel probe (specific to *C. cellulovorans*) had 9 mismatches to the sequence of *C. carboxidivorans* and 10 to the sequence of *C. acetobutylicum*, Clocar probe (specific to *C. carboxidivorans*) had 4 mismatches to *C. cellulovorans* and 6 to *C. acetobutylicum* sequences, and Cloace probe (specific to *C. acetobutylicum*) had 10 mismatches to *C. cellulovorans* and 11 to *C. carboxidivorans* sequences (Supplementary Fig. S2).

**Table 3:** Primer pair and TaqMan probes used within this study for multiplex qPCR assay.

Target organism	Name	Primer and probe sequence (5′–3′)	Amplicon size/probe position	Tm	GC content (%)
Bacterial 16S rRNA gene	ClosF	F—CGAAAGGGAGATTAATACCGCA	116 bp	58.28	45
	ClosR	R—GAGCCGTTACCTCACCAAC		58.16	58
*C. cellulovorans*	Clocel	Pr—VIC-CGCATGAGAGATGTATCAAAGGAGCAAT-BHQ1	37–64	65.2	43
*C. carboxidivorans*	Clocar	Pr—6FAM-TAAAGGAGTAATCCGCTTTGAGATGGGC-BHQ1	53–80	66.7	46
*C. acetobutylicum*	Cloace	Pr—Cy5-TGATTCTTGAGCCAAAGGATTTATTCGC-BHQ2	41–68	65.8	39

### Preparation of qPCR standards

Genomic DNA was extracted from cell pellets of pure cultures using the *Chellex* 100 resin (Bio-Rad, Spain) following the manufacturer’s instructions.

Standard DNA templates from *C. acetobutylicum* DSM 792*, C. carboxidivorans* DSM 15243 and *C. cellulovorans* DSM 3052 were prepared as circular plasmids. PCR products of the 16S rRNA genes were generated by conventional PCR using the 27F–1492R primer pair^42^, inserted into a pCR4-TOPO cloning plasmid using the TOPO TA Cloning Kit (Invitrogen, CA) and purified using the GeneJET Plasmid Miniprep Kit (Thermo Scientific, Spain), following the manufacturers’ instructions. The presence of the correct inserts was checked by sequencing (Macrogen Inc, Spain) from plasmid specific M13 F/R primers. The number of 16S rRNA gene copies for every standard was estimated from total DNA quantifications (Qubit 2.0, Thermo Fisher Scientific, USA), the expected size of plasmid + insert, and a mass of 650 g per mole bp. Ten-fold dilutions were prepared and used as standards ranging from 100 to 1 × 10^8^ copies/µL. For the multiplex qPCR assay, the three standard curves were mixed, resulting in a linear concentration range of 3.33 × 10^1^ to 3.33 × 10^7^ copies/µL for each *Clostridium* species. This standard curve was used for both the *Clostridium* and the total bacterial 16S rRNA gene quantification.

### Multiplex qPCR with TaqMan probes

Quantitative PCR (qPCR) was carried out using a Lightcycler 96 Real-Time PCR instrument. All reactions were performed in a final volume of 20 µL containing: 1 µL of DNA template (with a concentration around 10 ng/µL), 10 µL of FastStart Essential DNA Probes Master 2X (Roche Diagnostics, Germany), 6 µL of DEPC water, and 3 µL of Assay Mix containing the corresponding TaqMan probe and primer pair (with a final reaction concentration of 0.25 µM of probe and 0.90 µM of each primer). The amplification was done with one preincubation step at 95 °C for 5 min, followed by 45 cycles of a two-step amplification (denaturalization at 95 °C for 10 s, annealing and extension at 61 °C for 30 s). A non-template negative control was included in all PCR runs to check for external contamination or non-specific increases in the fluorescence. Samples were analysed in duplicates, and assays were repeated when variations in the Cq value were above 10%.

Efficiency and the linear range of quantification were calculated using a ten-fold dilution series from 3.33 × 10^1^ to 3.33 × 10^7^ copies/µL. Efficiency (*E*) was calculated using the equation: E = [10^(−1/slope)^ − 1], being 3.32 the slope for an 100% efficiency. Linear quantification range was obtained calculating the standard deviation (SD) between dilutions, accepting only those with a SD value lower than 0.34, and the limit of detection was calculated using eight replicas of the standard curves. Results were analysed with the Probit test using Minitab 14 Statistical Software (Pennsylvania, US) by plotting the ratio of positive/negative amplifications obtained against the amount of target genes present in each reaction, as described in Lopez-Siles et al.^43^.

### Specificity assay

To check for probes specificity, DNA extracts from different reference strains were used, 10 from species belonging to the *Clostridium* genus (*C. acetobutylicum* DSM 792, *C. carboxidivorans* DSM 15243, *C. cellulovorans* DSM 3052, *C. kluyveri* DSM 555*, C. drakei* DSM 12750, *C. leptum* DSM 753, *C. autoethanogenum* DSM 10061, *C. ljungdahlii* DSM 13528, *C. viride* DSM 6836 and *C. coccoides* ATCC 29236) and 4 other *Firmicutes* (*E. limosum* DSM20543, *L. plantarum* ATCC 14917, *L. mesenteroides* ATCC 8293 and *S. aureus* DSM 20231). To further check probe sensitivity, different DNA mixtures containing different amounts of the three targeted *Clostridium* species (ranging from 10 ng to 0.1 ng) were also analysed.

### Implementation of the different consortia

A total of three different consortia were tested, in triplicates: consortium 1, containing the cellulolytic *C. cellulovorans* and the solventogenic *C. acetobutylicum* and cellobiose as the carbon source; consortium 2, with the acetogenic *C. carboxidivorans* and the solventogenic *C. acetobutylicum* and glucose as the carbon source; and consortium 3, with the three *Clostridium* species and cellobiose as carbon source. The incubation of the different consortia was started with a 10% (v:v) inoculation of two or three (depending on the consortia) overnight cultures of the corresponding species. In all cases, cultures used for inoculation were not older than 24 h and were incubated in the same medium regardless of the organic carbon source, cellobiose for *C. cellulovorans*, and glucose for *C. carboxidivorans* and *C. acetobutylicum*. Incubations were performed in closed gas-tight serum bottles (nominal volume: 500 mL; working volume: 150 mL) at 37 °C ± 1 °C with constant agitation (130 rpm) in a Stuart SI500 incubator (Bibby Scientific Limited, OSA, UK). All three strains were also grown individually, following the same conditions, and used as controls.

### Monitoring the consortia dynamics

Liquid samples (2 mL) were taken at a regular basis for the first 24 h, and after 48 h and 96 h of the fermentation experiment, to monitor the changes in the consortium composition. Samples were centrifuged (10 min, 12,000 rpm), and pellets were kept at − 20 °C until DNA extractions were performed. DNA extracts were quantified using Qubit 2.0 Fluorometer (Thermo Fisher Scientific, USA) and kept at − 20 °C until qPCR analysis.

In addition, samples obtained at 0, 24, 48 and 96 h were filtered through a 0.22 µm pore-size filter and used for volatile fatty-acids (i.e., acetate, butyrate and caproate), alcohols (i.e., ethanol, acetone, butanol and hexanol) and sugars (i.e., cellobiose or glucose) determinations. Volatile fatty acids and alcohols were analysed by gas chromatography using an Agilent 7890A (Agilent Technologies, US) equipped with a DB-FFAP column and coupled to a flame ionization detector (FID). Acetone was measured by GC-FID with an Agilent 7840A equipped with an HP5 column following the method described in Fu et al.^44^. Glucose and cellobiose were quantified using an ionic chromatograph (Dionex ICS5000, USA) equipped with a Carbopac PA20 column and an electrochemical detector (ED). Gas phase analyses were performed only at the end of the experiment to analyse residual CO_2_ accumulation. Gas pressure was measured using a digital pressure sensor (Testo 512, Spain). A gas sampling syringe (5 mL) was used to collect samples from the headspace. Gas composition (CO_2_, H_2_, O_2_ and N_2_) was analysed by gas chromatography using a 490 Micro GC system (Agilent Technologies, USA).

### Testing the reliability of the quantification with general established methods

To check the accuracy of the quantification, the total bacterial 16S rRNA gene was also quantified using the primer pair 341F–534R specific for *Eubacteria*^37^ with SYBR Green I Master Mix (Roche Life Science, Switzerland). Eventually, a barcoded amplicon sequencing protocol directed to the 16S rRNA gene using 515F – 806R primer set was used to check for the presence of contamination in long fermentation runs, and to estimate the proportion of every consortium member during growth.

### Supplementary Information

Supplementary Information.

## Data Availability

Sequences obtained in this study have been submitted to the GenBank database under the SRA accession number PRJNA981705.
