# Supplementary material for: Design and validation of a multiplex PCR method for the simultaneous quantification of Clostridium acetobutylicum, Clostridium carboxidivorans and Clostridium cellulovorans
Source: Sci Rep. 2023 Nov 16;13:20073. doi: 10.1038/s41598-023-47007-w (PMC10654501; doi:10.1038/s41598-023-47007-w)
Supplement: Supplementary file 1 — Supplementary Information. [file 41598_2023_47007_MOESM1_ESM.pdf]

Design and validation of a multiplex PCR method for the simultaneous quantification of *Clostridium acetobutylicum, Clostridium carboxidivorans* and *Clostridium cellulovorans*

Feliu-Paradeda, Laura[1], Puig, Sebastià[2] and Bañeras, Lluis[1*]

Supplementary Figures

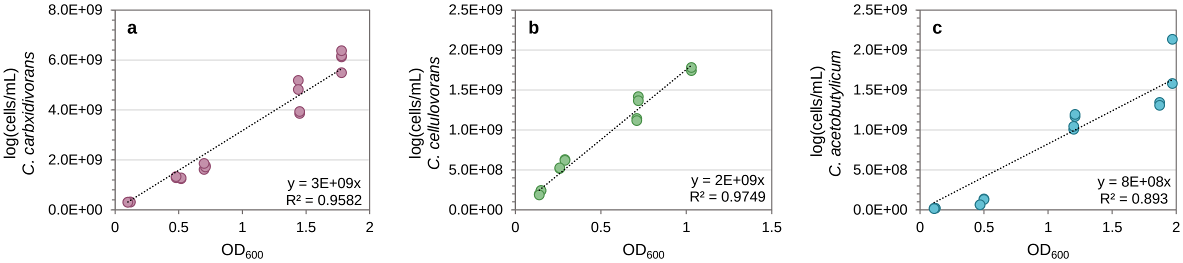

Supplementary Figure S1. Optical density (OD_{600}) versus cell concentrations obtained using qPCR (log (cells/mL) plot for A) *C. carboxidivorans*, B) *C. cellulovorans* and C) *C. acetobutylicum* mono-cultures.

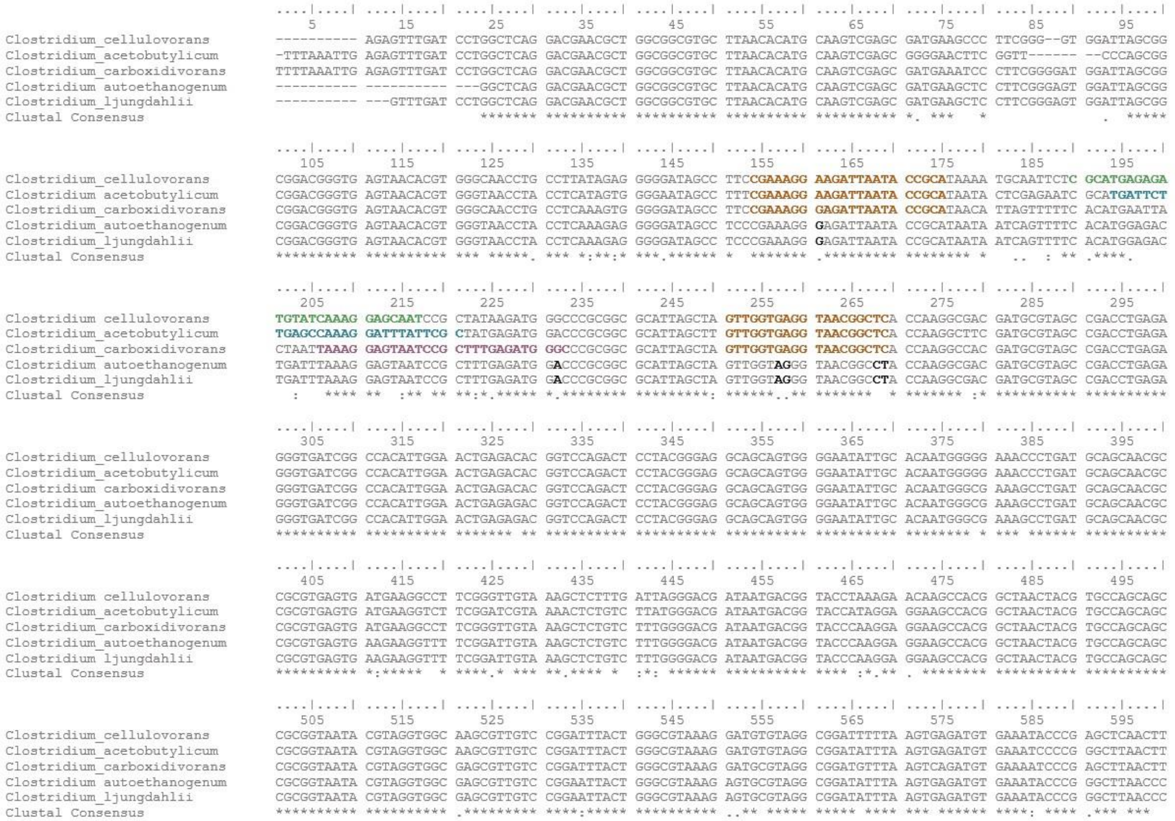

Supplementary Figure S2. 16S rRNA gene alignment of *C. cellulovorans* 743B*, C. acetobutylicum* ATCC 824*, C. carboxidivorans* P7, *C. autoethanogenum* DSM 10061 and *C. ljungdahlii* DSM 13528. Primer pair location (forward and reverse) are marked in orange; Clocel probe in green; Cloace probe in blue; and Clocar probe in magenta. Differences in the sequence between primer pair and Clocar probe with both *C. autoethanogenum* and *C. ljungdahlii* sequences are highlighted in black.

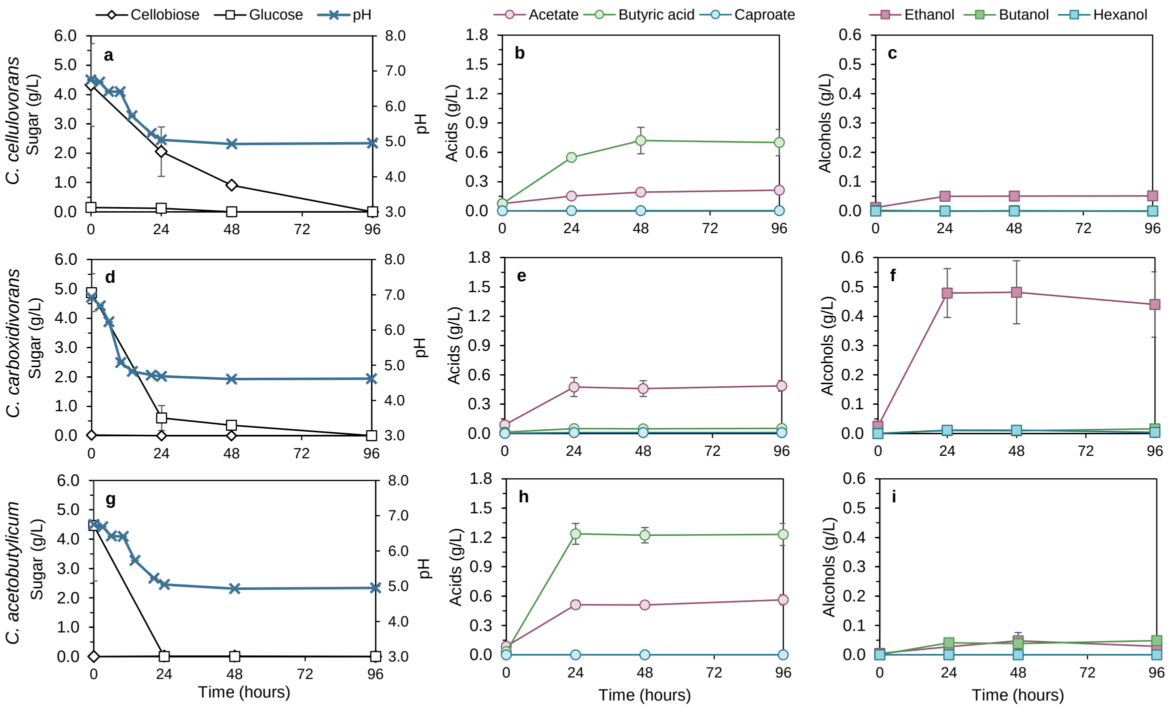

Supplementary Figure S3. Sugar, pH, acids and alcohol concentration in *C. cellulovorans* (A, B, C), *C. acetobutylicum* (D, E, F) and *C. carboxidivorans* (G, H, I) mono-cultures.

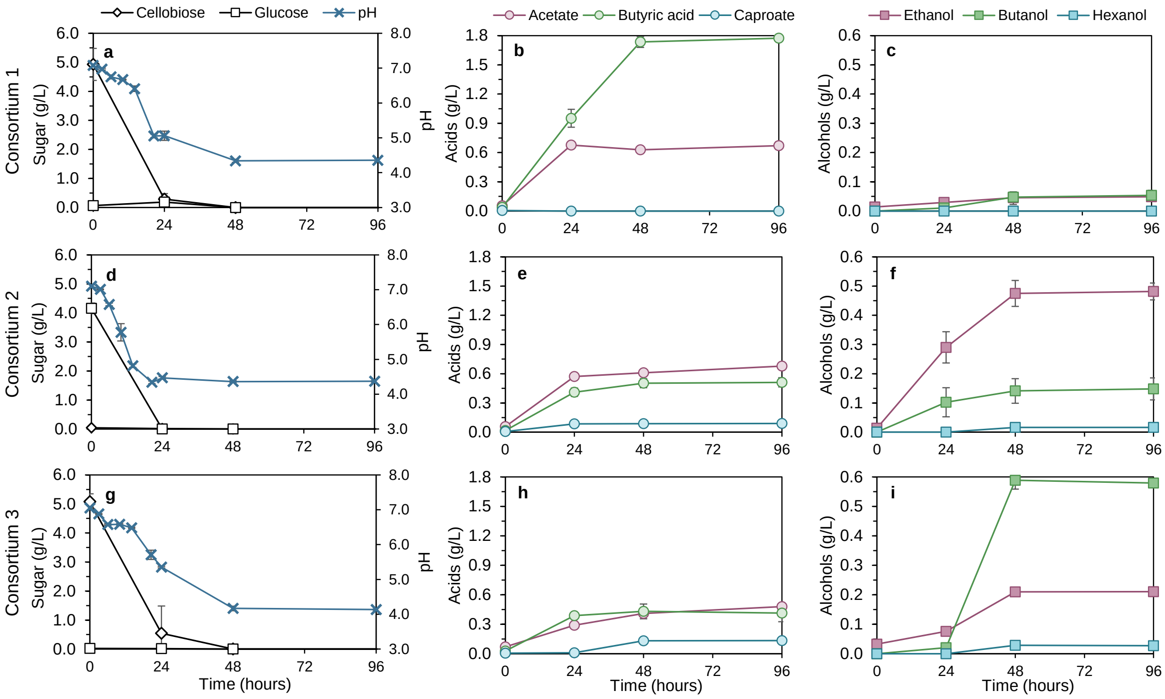

Supplementary Figure S4. Sugar, pH, acids and alcohol concentration in consortia 1 (A, B, C), 2 (D, E, F) and 3 (G, H, I).

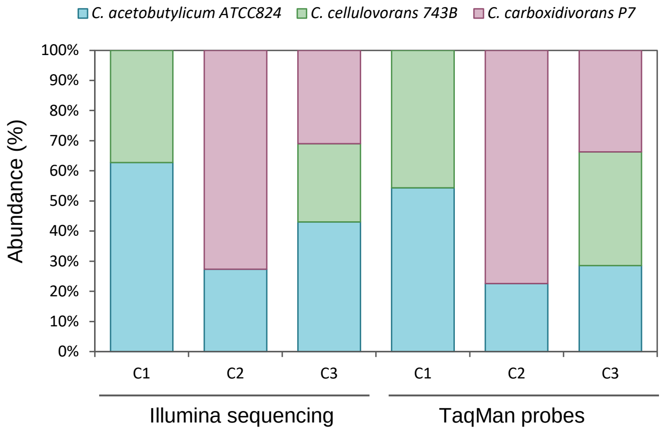

Supplementary Figure S5. *Clostridium* species abundance within each consortium through analysing the community composition by Illumina sequencing, or using the TaqMan probes designed in this study. C: consortium.

Supplementary Tables

Supplementary Table S1. Acids, alcohols and acetone concentration (in mmols C/L), gas pressure and CO_2 produced (mmols) at the end of the 4-day batch fermentation experiment for the three mono-cultures and consortia tested. N = 3, ± SD.

Culture type	*Clostridium* spp.	End products (mM C)							Gas phase parameters	
		Acetic acid	Butyric acid	Caproic acid	Ethanol	Acetone	Butanol	Hexanol	Pressure (Bar)	CO_2 (mmols)
Mono-cultures	*C. carboxidivorans*	16.25 ± 1.82	2.36 ± 0.19	0.52 ± 0.20	19.11 ± 3.85	-	0.88 ± 0.38	0.24 ± 0.11	0.51 ± 0.00	0.31 ± 0.00
	C. acetobutylicum	18.75 ± 1.70	55.94 ± 5.12	-	1.26 ± 0.37	0.62 ± 0.16	2.59 ± 0.65	-	0.85 ± 0.04	1.23 ± 0.11
	C. cellulovorans	7.12 ± 0.93	31.78 ± 6.06	-	-	-	-	-	0.63 ± 0.03	0.63 ± 0.09
Consortium 1	*C. acetobutylicum*<br>*C. cellulovorans*	22.36 ± 1.24	80.46 ± 1.36	-	2.13 ± 0.48	0.59 ± 0.1	2.88 ± 0.96	-	1.01 ± 0.23	1.48 ± 0.73
Consortium 2	*C. acetobutylicum*<br>*C. carboxidivorans*	22.61 ± 0.63	23.21 ± 2.15	4.62 ± 1.52	20.92 ± 1.25	0.37 ± 0.11	7.99 ± 2.05	0.95 ± 0.18	0.87 ± 0.02	1.30 ± 0.05
Consortium 3	*C. acetobutylicum*<br>*C. cellulovorans*<br>*C. carboxidivorans*	15.98 ± 0.48	18.80 ± 4.03	6.85 ± 0.92	9.13 ± 0.68	0.24 ± 0.13	31.27 ± 0.57	1.60 ± 0.16	0.89 ± 0.03	1.34 ± 0.09
